# Capillary nano-immunoassay for Akt 1/2/3 and 4EBP1 phosphorylation in acute myeloid leukemia

**DOI:** 10.1186/1479-5876-12-166

**Published:** 2014-06-12

**Authors:** Himalee Sabnis, Heath L Bradley, Silvia T Bunting, Todd M Cooper, Kevin D Bunting

**Affiliations:** 1 Department of Pediatrics, Aflac Cancer and Blood Disorders Center, Emory University, 1760 Haygood Drive NE, Atlanta, Georgia, USA; 2 Children’s Healthcare of Atlanta, 1405 Clifton Road, Atlanta, Georgia, USA

**Keywords:** Nano-immunoassay, Biomarker, Leukemia, Capillary electrophoresis, mTOR

## Abstract

**Background:**

Overall cure rates in acute myeloid leukemia (AML) continue to range between 60-65% with disease relapse being a major cause of mortality. The PI3K-Akt-mTOR kinase pathway plays a vital role in pro-survival signals within leukemic cells and inhibition of this pathway is being investigated to improve patient outcomes. Tracking activation of multiple signaling proteins simultaneously in patient samples can be challenging especially with limiting cell numbers within rare sub-populations.

**Methods:**

The NanoPro 1000 system (ProteinSimple) is built on an automated, capillary-based immunoassay platform and enables a rapid and quantitative analysis of specific proteins and their phosphorylation states. We have utilized this nano-immunoassay to examine activation of Akt 1/2/3 and downstream mTOR target - eukaryotic initiation factor 4E-Binding Protein 1 (4EBP1).

**Results:**

Assays for Akt 1/2/3 and 4EBP1 were standardized using AML cell lines (MV4-11, MOLM-14, OCI-AML3 and HL-60) prior to testing in patient samples. Target inhibition was studied using mTOR 1/2 inhibitor AZD-8055 and results were corroborated by Western blotting. The assay was able to quantify nanogram amounts of 4EBP1 and Akt 1/2/3 in AML cell lines and primary pediatric AML samples and results were quantifiable, consistent and reproducible.

**Conclusion:**

Our data provides a strong basis for testing this platform on a larger scale and our long term aim is to utilize this nano-immunoassay prospectively in de-novo AML to be able to identify poor responders who might benefit from early introduction of targeted therapy.

## Introduction

Acute myeloid leukemia (AML) affects 16,000 -18,000 people annually in the United States and approximately 75% will succumb to the illness [1]. 6% of all patients affected are under the age of 20 years [1]. In spite of the advances made in the treatment of acute myeloid leukemia with chemotherapy as well as hematopoietic stem cell transplantation, overall cure rates remain at 60-65% with relapse being a major cause of mortality [2]. Of those relapsed patients, only a third are salvageable with current treatment regimens [3,4]. Discovery of both cytogenetic and molecular abnormalities in AML has resulted in the development of the current prognostic sub-groups in AML [5] and the molecular abnormalities play an important role in leukemogenesis, especially in patients with normal cytogenetics [6].

Downstream of these molecular aberrations in leukemic cells, highly complex and inter-linked networks of signaling pathways control cell survival growth, proliferation, self renewal and differentiation. Up-regulation of the PI3K-Akt-mTOR (PI3K-Akt-mammalian target of rapamycin) pathway occurs via mutations in surface receptors like FLT3, c-Kit or by mutations in the genes encoding pathway constituents like PI3K, PTEN or Akt [7,8] . Akt is a serine/threonine protein kinase that exists in three conserved isoforms: Akt 1, 2 and 3. Of the three iso-forms present, Akt 1 and 2 are expressed to a higher extent in hematopoietic stem cells [9]. Akt is phosphorylated at Thr 308 by up-stream phosphoinositide-dependent protein kinase 1 (PDK-1) and at Ser 473 by mTOR complex 2 (mTORC2). Akt plays an important role in key cellular processes such as protein translation, cell proliferation, cell cycle, and apoptosis through its multiple downstream targets however activating mutations in Akt have not been described in AML [10] . Akt can be constitutively phosphorylated in AML which results in depletion of normal hematopoietic stem cells [11].

Activation of the mTOR pathway is seen in up to 80% of AML patients and is associated with a shortened overall survival. mTOR kinase is also a serine/threonine kinase that complexes with other proteins [12]. mTORC2 mainly functions to phosphorylate and activate Akt whereas mTORC1 plays a central role in the translational machinery of normal and leukemic cells via its downstream targets - p70S6 Kinase and eukaryotic initiation factor (eIF) 4E binding protein-1 (4EBP1) [12,13]. p70S6 Kinase phosphorylates the 40S ribosomal subunit protein S6 and thereby allows translation of proteins involved in cell growth and hypertrophy. 4EBP1 phosphorylation results in release of the inhibition of eIF4E and enables the formation of eIF4F complex. This complex is necessary for the cap-dependent translation of highly structured mRNAs which encode genes such as c-Myc, Mcl-1 and VEGF that are involved in cell survival [13]. In certain subtypes of AML (FAB M4/M5) eIF4E itself has been shown to function as an oncogene via transcriptional up-regulation by nuclear factor kappa-light-chain-enhancer of activated B cells (NF-κB) [14]. Both p70S6 Kinase and 4EBP1 are downstream targets of mTOR however, inhibition of 4EBP1 phosphorylation is key for ensuring efficacy of mTOR antagonists [15]. Thus inhibiting downstream mTOR targets has played a prominent role in anti-leukemic therapy for several years and continues to be an active area of research [12].

Molecular differences in Akt-mTOR pathway with AML patients may provide key information to better define the pathogenesis of disease, especially in patients with normal cytogenetics. Traditionally, techniques such as Western Blot and intra-cellular flow cytometry have been used for this purpose but these have several limitations - they require large number of cells, require technical expertise and quantitative results are difficult to obtain. The NanoPro 1000 system (ProteinSimple) enables a rapid and quantitative analysis of specific proteins from small quantities of sample (dependent on cell size and percentage of protein). The NanoPro provides precise and quantitative data of the multi-site phosphorylation states of a specific protein of interest. This degree of phospho-protein specificity and rapid turn-around time are unique. The system is built on an automated, capillary based immunoassay platform. Proteins are separated by isoelectric focusing separation (PI) to resolve the various modification states of proteins, immobilized, and probed with specific antibodies. Signal intensity is detected by a horseradish peroxidase-conjugated chemiluminescence system and the data is shown as an electropherogram. It can examine numerous proteins within few cells using the same antibodies used in traditional Western blots. This feature of the NanoPro 1000 makes it a unique tool to look at signaling within rare leukemic cell populations as it requires very small amounts (as low as 80 ng) of protein per capillary. It is also capable of simultaneously detecting multi-phosphorylation states of the same protein which is impossible to do by Western Blot or intracellular flow cytometry. We used the NanoPro 1000 platform on AML cell lines to standardize the assays for 4EBP1 and Akt 1/2/3.

## Methods

### Cell lines

MV4-11, MOLM-14, OCI-AML3 and HL-60 cell lines were used for this analysis. Cell lines were grown in RPMI (MOLM-14), alpha-MEM (OCI-AML3) or IMDM (MV4-11 and HL-60) and supplemented with 10% or 20% fetal bovine serum with 1% penicillin-streptomycin. Cell density was maintained at 0.1 million cells/ml and cultures were split every 48 hours maintaining cell viability. Cells were harvested when confluent at 48 hours for analysis of baseline signal using the NanoPro 1000 assay as outlined below. Cell lines were treated for 24 hours in the presence of specific inhibitors prior to analysis.

### Primary patient samples

Primary AML bone marrow samples were obtained from the Children’s Oncology Group (COG) Myeloid Diseases Reference Laboratory on pilot study protocol AAML12B13. The study was approved by Emory Institutional Review Board and the COG Myeloid Diseases Committee. These bone marrow samples were obtained from pediatric AML patients after written informed consent at the time of diagnosis and were de-identified prior to storage. Samples were ficolled to isolate mono-nuclear cells and frozen with 10% DMSO. Information provided to the investigators included age, gender, sample mono-nuclear cell count and FLT3 mutation status. Samples were thawed at 37°C and placed in RPMI media supplemented with 20% fetal bovine serum and 100 ng/ml cytokines (IL-3, G-CSF, GM-CSF, SCF – Gemini Bio Products Cat. #300-151P, #300-123P, #300-124P and #300-185P respectively) for 24 hour drug effects. For baseline analysis, samples were analyzed immediately after thawing.

### Drug treatment

AZD-8055 was obtained from Chemietek Biochemicals (Cat. No. CT-A8055). It was dissolved in DMSO and stored at -20 C. Both cell lines and primary AML samples were treated with AZD-8055 with concentrations ranging from 25–1000 nM for varying times to demonstrate target inhibition.

### Western blotting

MV4-11 cells (5×10^6^ cells) were treated for 1 hour with AZD-8055 at concentrations ranging from 25–1000 nM and with vehicle DMSO. Cell pellets were lysed in 150 μl Bicine Chaps lysis buffer (containing protease and phosphatase inhibitor cocktail made as per Protein Simple specifications). Protein concentrations were determined by Bio-Rad protein assay. Proteins were separated using SDS-polyacrylamide gels, transferred to polyvinylidene diflouride membranes (EMD Millipore) and blocked in 5% non-fat dry milk. Primary antibody incubations were performed overnight at 4°C, followed by incubation in secondary horseradish peroxidase-linked anti-rabbit or anti-mouse secondary antibody at room temperature for 1 hour. Primary antibodies used were total 4EBP1 (Cell Signal Cat #9644 s), phospho-serine 65 4EBP1(Cell Signal Cat #9451 s), phospho-threonine 37/46 4EBP1 (Cell Signal Cat #2855), total Akt 1/2/3 (Santa Cruz Cat #sc-8312), β-2 microglobulin (Abcam Cat #ab75853) and β-actin (Sigma Cat #A5441). These were used at a concentration of 1:1000 except for β-actin (conc. 1:10,000) and secondary antibodies (Anti-mouse Cell Signal Cat #7076S, Anti-rabbit Cell Signal Cat #7074S) were used at a concentration of 1:2000.

### Nano-immunoassay

All isoelectric separations were performed on the NanoPro 1000 (ProteinSimple, Santa Clara, CA) by mixing 1 part lysate with 3 parts of ProteinSimple’s Generation 2 pH 5–8 (nested) separation gradient which contains a pH 2–4 plug (Cat #040–972). Standard pI Ladder 3 (ProteinSimple Cat #040–646) supplemented with individual pI Standard 5.5 (ProteinSimple Cat #040–028) diluted 60 fold was added to the ampholyte pre-mix. Lysates were then separated for 40 min at 21,000 μW in individual capillaries. After separation the proteins in the lysate were immobilized to the capillary wall by subjecting them to UV exposure for a period of 80 seconds. After two washes of 150 seconds each, primary antibodies were introduced into the capillaries for a period of 2 hours. Antibodies for 4EBP-1 were used at a 1:25 dilution, whereas antibodies for AKT 1/2/3 and β-2 Microglobulin were used at 1:100 dilutions. After another two washes of 150 seconds each, samples were run either with or without amplification reagents. Secondary anti-rabbit-HRP-conjugated antibodies (ProteinSimple Cat #040–656) or secondary anti-rabbit-biotin-conjugated antibodies (ProteinSimple’s amplified rabbit secondary antibody kit - Cat #041–126) were loaded into the capillary for 1 hour. Amplification was performed only for 4EBP1 antibodies using primary patient samples and AML cell lines. After a third set of two washes of 150 seconds each, either streptavidin, conjugated with horse radish peroxidase (ProteinSimple Cat #041–126), or antibody diluent was loaded into the capillary for 2 hours or 10 minutes respectively. After a final two wash cycle of 150 seconds each, a luminol-peroxidase 1:1 mix (ProteinSimple Cat #040–0652 and 040–684) was flowed through the capillaries and chemiluminescence was detected at 30, 60, 120, 240, 480, and 960 seconds. Primary 4EBP1 antibodies used were similar to those used for Western blotting and in addition rabbit polyclonal total Akt1/2/3 (Santa Cruz Cat #sc-8312) was used for the assay. To determine phospho-peaks, sample lysates were pre-treated with 100 U lambda phosphatase or vehicle according to the manufacturer’s instructions (Millipore, Cat #14-405). Lysates were incubated in 1× DTT-containing lambda phosphatase buffer for 1 hour at 37°C before running on the NanoPro.

### Statistical analysis

All data was derived as a result of three independent experiments, unless stated otherwise. Two tailed t-test was used to calculate p-values and values less than 0.05 were considered to be significant.

## Results

### The NanoPro 1000 platform can be used to measure 4EBP1 phosphorylation within AML cell lines and to demonstrate target inhibition

**Figure 1:**
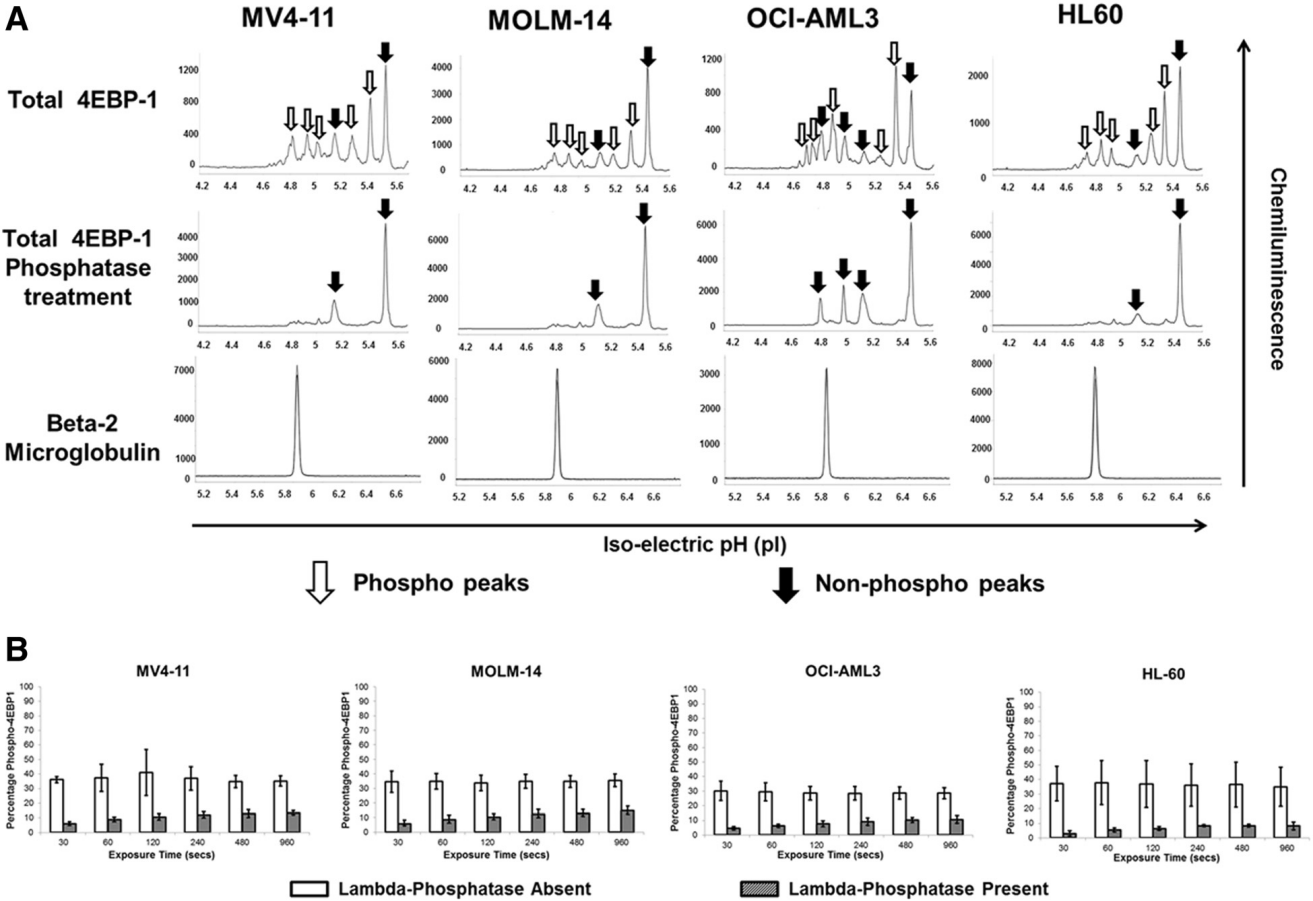
**Measurement of total 4EBP1 in AML cell lines. A)** Electropherogram depicting levels of total and phosphorylated 4EBP1 in AML cell lines. AML cell lines MV4-11, MOLM-14, OCI-AML3 and HL60 were analyzed at baseline for expression of 4EBP1. 80 ng of protein was used for analysis. β-2 Microglobulin was used as loading control. Total 4EBP1 antibody detects both phosphorylated and non-phosphorylated protein. Samples were treated with lambda-phosphatase or reaction buffer alone and decrease in phosphorylation was noted. X-axis represents iso-electric pH and y-axis represents luminescence units. **B)** Change in phosphorylation was measured at exposure times varying between 30–960 secs after phosphatase treatment in all cell lines. The experiments were performed in triplicate (*p < 0.05).

**Figure 2:**
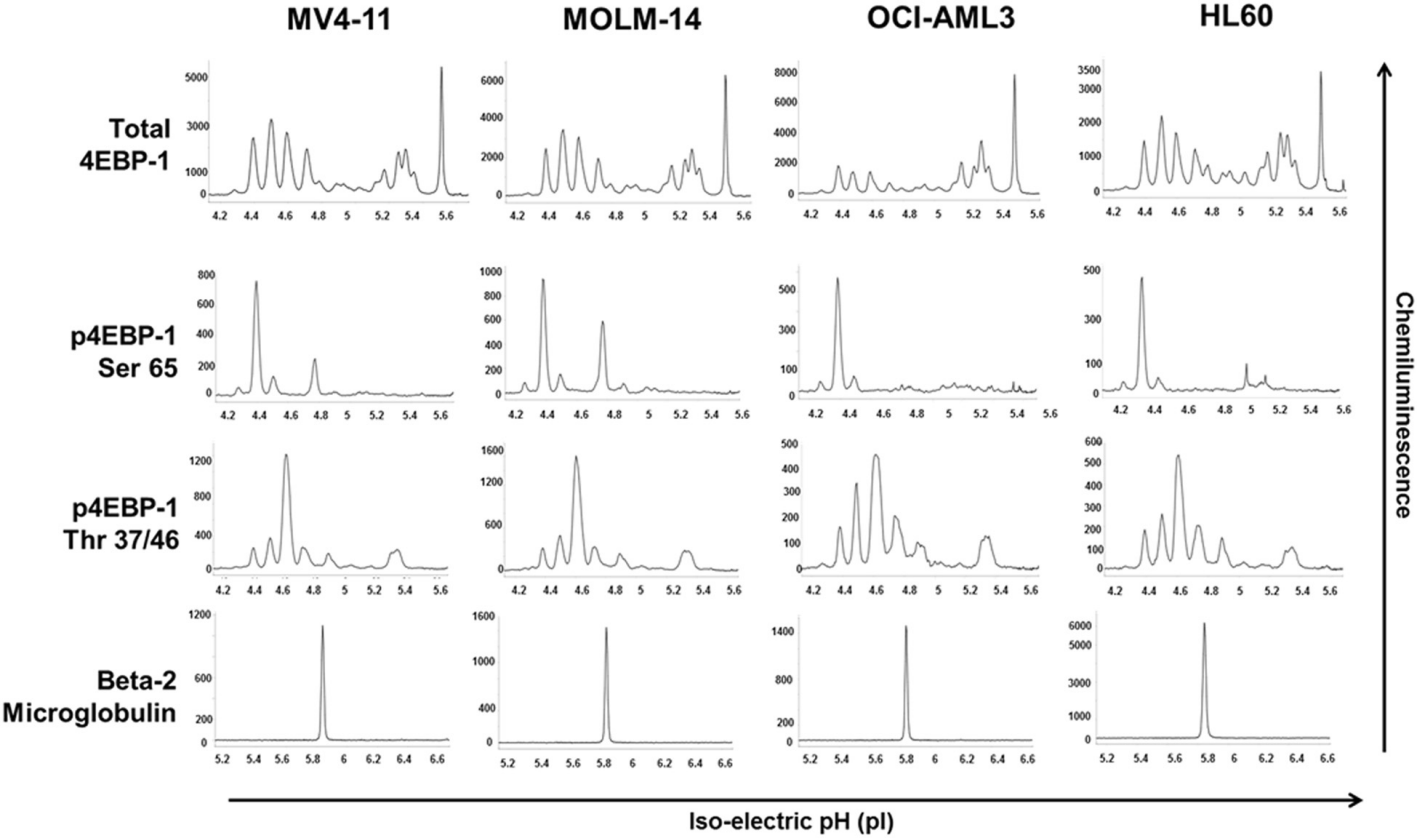
**Measurement of phosphorylated 4EBP1 within AML cell lines.** AML cell lines MV4-11, MOLM-14, OCI-AML3 and HL60 were analyzed at baseline using total 4EBP1, phospho-specific Serine 65 and Threonine 37/46 4EBP1 antibodies. β-2 Microglobulin was used as loading control.

**Figure 3:**
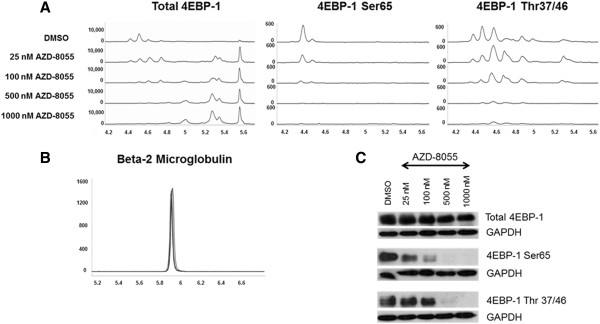
**Use of 4EBP1 assay to examine target inhibition within MV4-11 cell line.** MV4-11 cells were treated with mTOR1/2 inhibitor AZD-8055 (25 – 1000 nM) for one hour and inhibition of 4EBP1 phosphorylation was noted using the NanoPro 1000 and Western blotting. **A)** Electropherogram shows the decrease in signal using both total and phospho-specific antibodies. **B)** β-2 Microglobulin was used as loading control. X-axis represents iso-electric pH and y-axis represents luminescence units. **C)** Western blotting confirms the same results with GAPDH as loading control.

We used AML cell lines to standardize assays for the NanoPro 1000. AML cell lines were analyzed at baseline for determination of total and phosphorylated forms of 4EBP1 (Figures 1 and 2). The pattern of 4EBP1 activation varied across cell lines. The total antibody was capable of detecting both phosphorylated and non-phosphorylated forms of the protein as depicted by the electropherogram tracing using the total 4EBP1 antibody (Figure 1-A). All samples were also treated with lambda phosphatase and analyzed simultaneously to show suppression of phosphorylation and an increase in the amount of un-phosphorylated protein. β-2 microglobulin was used as a loading control. We used the area-under-curve (AUC) for the total 4EBP1 antibody to calculate the percentage of phosphorylated forms of 4EBP1 in the AML cell lines (Figure 1-B). Each line demonstrated different degrees of phosphorylation – MV4-11 (36.9%), MOLM-14 (34.7%), OCI-AML3 (29%) and HL60 (36.6%). Treatment with lambda phosphatase resulted in a decreased percentage of phosphorylated forms of the protein to 10.6% in MV4-11, 11.1% in MOLM-14, 8.1% in OCI-AML3 and 6.7% in HL60 cells and these differences were statistically significant (p-value <0.05). Interestingly, OCI-AML3 cells showed a unique peak pattern from the other 3 cell lines with 2 additional peaks that were resistant to lambda phosphatase treatment. The reasons for this are unknown but may be related to isoform specific expression. This difference highlights the importance of validating phospho-peaks in every cell line or patient sample.Although the treatments in Figure 1 were well controlled for the lambda phosphatase buffer and incubation time, the treatment conditions alone modified the pI of the peaks relative to un-manipulated samples shown in Figure 2 (compare total 4EBP1 peaks). Sample lysates not treated with lambda phosphatase or vehicle had the same number of peaks but the isoelectric points differed. This does not affect the ability of the assay to accurately determine the percentage of phosphorylated protein, however, to further test antibodies that are specific to phosphorylated 4EBP1 we tested additional antibodies. Serine 65 4EBP1 antibody is specific for the phosphorylation site and showed a single peak profile within each cell line that varied between 4.5 - 4.7 within cell lines. The Threonine 37/46 antibody was able to detect site specific phosphorylation but also demonstrated capability of detecting non-phosphorylated forms as evident from the electropherogram tracing, although the signal of the phosphorylated peaks was much higher. The intensity of signal using phospho-specific antibodies was much lower than the signal detected by the total 4EBP1 antibody (reasons not completely understood). β-2 microglobulin was used as a loading control.In order to validate the ability of this platform to detect specific target inhibition, we treated MV4-11 cells with specific mTOR 1/2 inhibitor AZD-8055 (25–1000 nM) for 1 hour. Cell lysates were obtained and analyzed simultaneously by Western blotting and by nano-immunoassay (Figure 3). The nano-immunoassay was performed using 80 ng of protein and was able to demonstrate a dose dependent decrease in phosphorylation with increasing concentrations of the drug as expected using total and phospho-specific 4EBP1 antibodies (Figure 3-A). Treatment with AZD-8055 resulted in a shift of the peak profile for the total antibody. The more acidic peaks denoting phosphorylated protein were reduced and there was a compensatory increase in the non-phosphorylated more basic peaks. The phospho-specific Ser 65 and Thr 37/46 antibodies showed a dose dependent decrease in the phosphorylation with a decrease in the acidic peak profile. β-2 microglobulin was used as a loading control for the nano-immunoassay and showed even loading across all samples (Figure 3-B). Western blotting performed using 10 μg of protein demonstrated a similar pattern with decreased phosphorylation with increasing concentration of AZD-8055 (Figure 3-C).

### Total Akt 1/2/3 antibody can be used to measure total and phosphorylated forms using nano-immunoassay in AML cell lines

**Figure 4:**
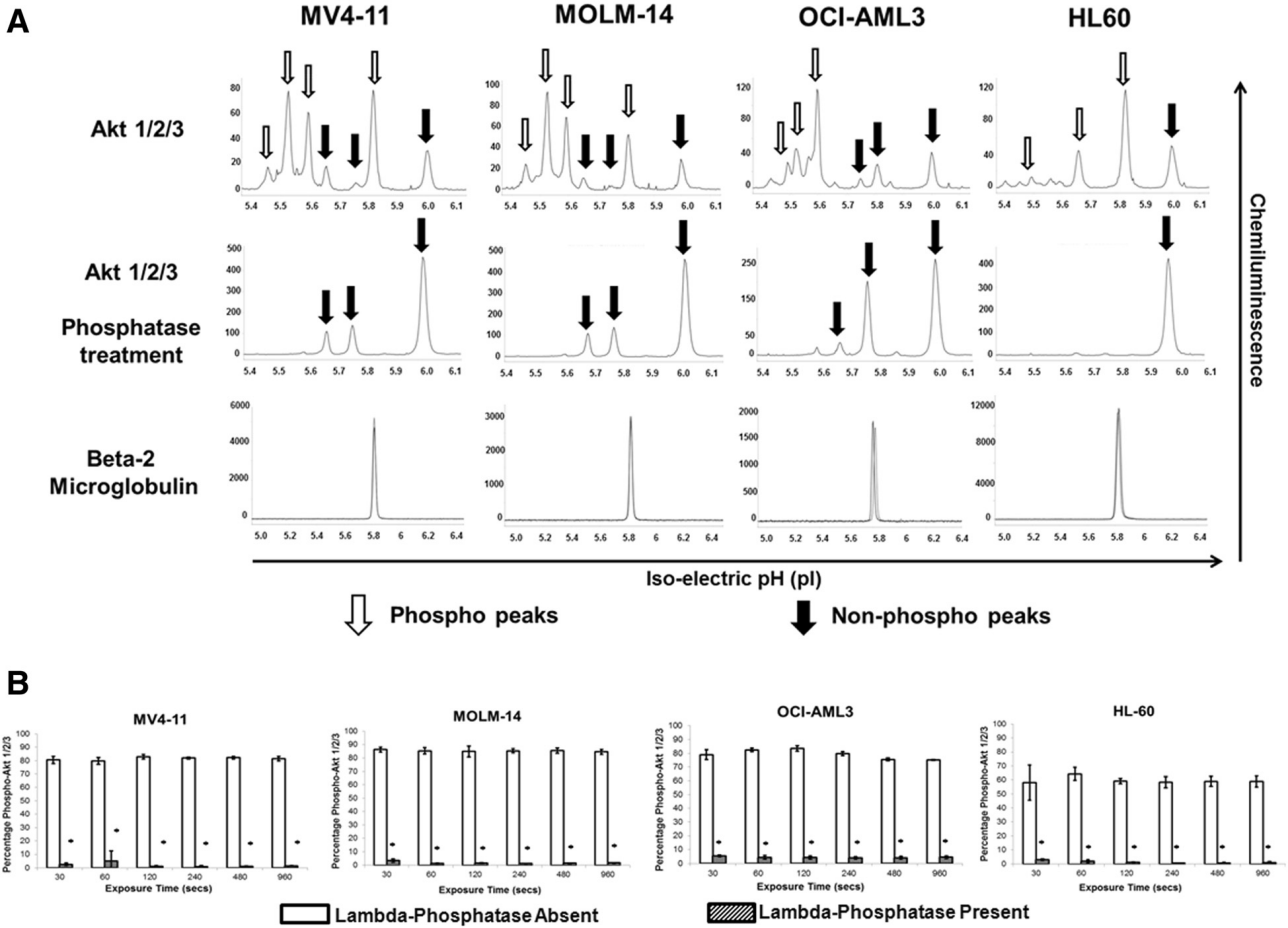
**Measurement of total and phosphorylated Akt 1/2/3 in AML cell lines. A)** Electropherogram depicting levels of total Akt 1/2/3 in AML cell lines. AML cell lines MV4-11, MOLM-14, OCI-AML3 and HL60 were analyzed at baseline for activation of Akt. 80 ng of protein was used for analysis. β-2 Microglobulin was used as loading control. Total Akt antibody detects both phosphorylated and non-phosphorylated protein which is demonstrated on treatment with phosphatase. X-axis represents iso-electric pH and y-axis represents luminescence units. **B)** Different AML cell lines exhibit different levels of Akt phosphorylation as demonstrated using total Akt 1/2/3 antibody and the phosphorylation is diminished on treatment with lambda phosphatase. The experiments were performed in triplicate (*p < 0.05).

Similar to 4EBP1 protein, we standardized the nano-immunoassay in AML cell lines for Akt 1/2/3 antibody. The total Akt 1/2/3 antibody was used which was capable of detecting both phosphorylated and non-phosphorylated forms. Specific phospho-Akt antibody assay is currently not standardized in our lab on the nano-immunoassay platform. AML cell lines were analyzed at baseline for expression of Akt 1/2/3 (Figure 4). Akt 1/2/3 expression and activation varied with all cell lines (Figure 4-A). In order to determine the specificity of the more acidic peaks for the phosphorylated protein, each line was treated with lambda-phosphatase concurrently and the electropherogram pattern revealed complete suppression of the phosphorylated peaks as expected and increase in the non-phosphorylated protein peaks. Our data showed expression of Akt 1 and 2 as expected in hematopoietic cells and was consistent with recently reported identification of the isoforms of the protein [16]. The non-phosphorylated forms of Akt 1 are detected in the pI range 5.6 – 5.8 and the non-phosphorylated form of Akt 2 is present in the 5.9 – 6.0 pI range. From the electropherogram it appears as though Akt 2 rather than Akt 1 is the predominant isoform in HL60 cell line and Akt 1 is present in higher quantities in MV4-11, MOLM-14 and OCI-AML3 cell lines. β-2 microglobulin was used as a loading control and demonstrated equal loading in both phosphatase-treated and non-treated samples. Results were further analyzed by AUC to determine the degree of phosphorylation within each cell line (Figure 4-B). Akt 1/2 was found to be phosphorylated similarly in MV4-11 (81.5%), MOLM-14 (85.2%) and OCI-AML3 (79.2%) cell lines and phosphorylated to a lower degree in HL60 (59.6%) cell line. Although the cell lines differed in the degree of Akt 1/2 activation /phosphorylation, treatment with phosphatase showed a significant decrease in the AUC for the phosphorylated peaks on phosphatase treatment to 2.3% in MV4-11, 1.8% in MOLM-14, 4.6% in OCI-AML3 and 1.5% in HL60 cell lines with the differences being statistically significant (p < 0.05).

**Figure 5:**
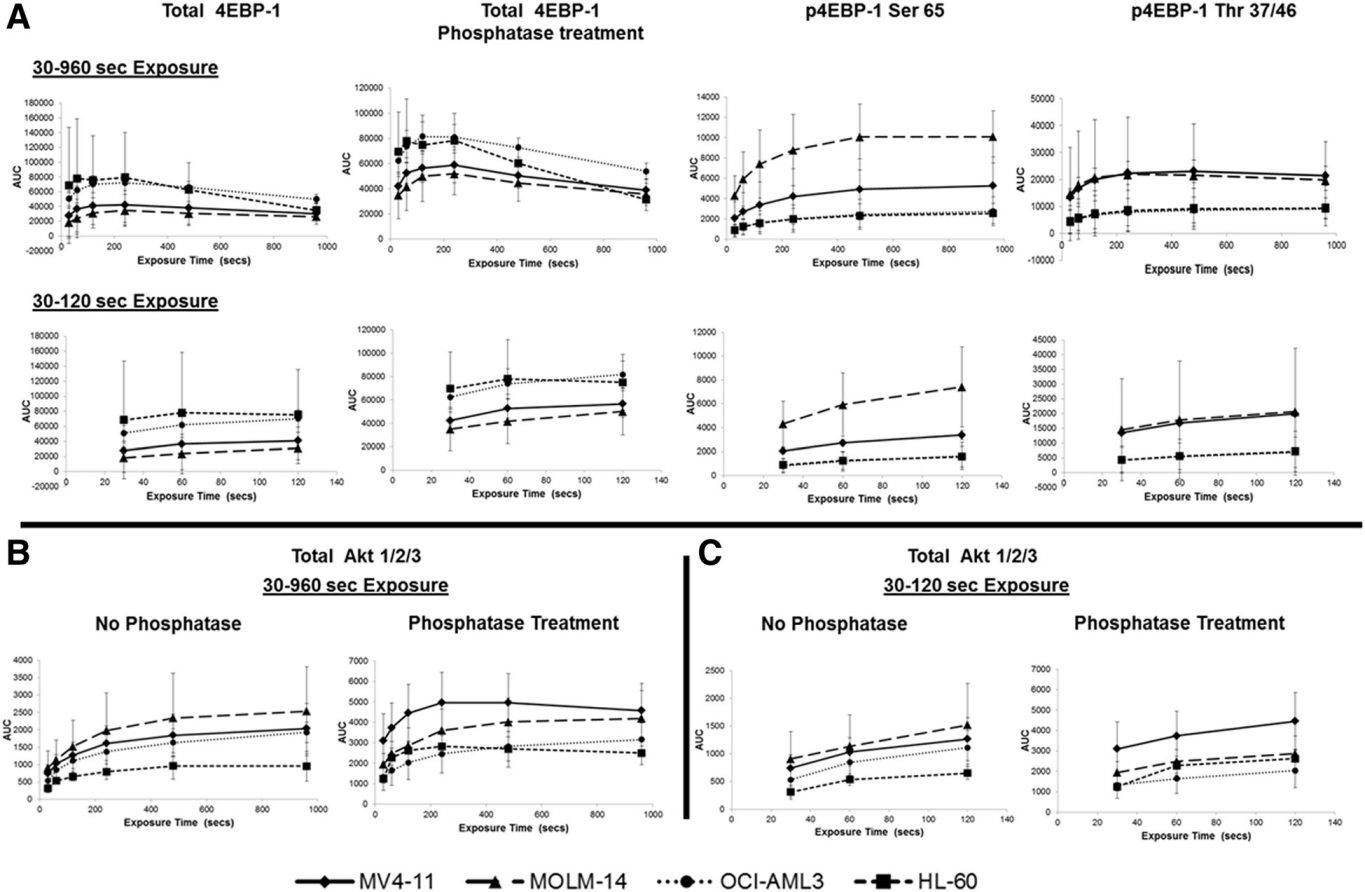
**Exposure time and protein amount is important for accurate measurement of signal.** The intensity of signal (AUC) for total and phosphorylated 4EBP1 **(A)** and total Akt 1/2/3 **(B & C)** was examined at exposure times between 30–960 seconds in MV4-11, MOLM-14, OCI-AML3 and HL60 cells. Higher exposure times (240–960 secs) resulted in signal burn-out which was not observed at lower exposure times (30–120 secs). More abundant proteins such as 4EBP1 exhibit improved linearity in signal with titration of protein loading (Additional file 1: Figure S1).

In order to demonstrate linearity of the signal intensity for total and phosphorylated 4EBP1 and total Akt, we plotted signal intensity against exposure time ranging from 30–960 secs for each antibody. The assay was run in triplicate for each cell line and results are shown in Figure 5. 4EBP1 antibodies are depicted in Figure 5-A and total Akt 1/2/3 antibody is depicted in Figure 5-B & C. The linearity of the signal was dependent on the duration of exposure and the abundance of the protein within the sample as demonstrated by the pattern for each antibody. At higher exposure times (240–960 secs), the signal for all antibodies exhibited a logarithmic increase in intensity and burn-out at high intensity. From data obtained in the baseline runs, it was evident that less than 40% of 4EBP1 is phosphorylated within these cell lines. In samples where the protein of interest was low (eg. Phospho-4EBP1), lower exposure times (30–120 secs) resulted in a linear increase (R-square value >0.94) in signal intensity, as was evident for both Serine 65 and Threonine 37/46 antibodies using 80 ng of protein per capillary. For proteins that are present in higher amounts such as total Akt 1/2/3, lower exposure times (between 30–120 secs) exhibited a less linear increase in signal intensity (R-square values between 0.8-0.99) and more abundant proteins such as 4EBP1 exhibited even less linear increases with R-square values between 0.2-0.9. To test the assumption that amount of protein was important for signal linearity, we repeated the total 4EBP1 assay by further titrating protein loading to 40 and 20 ng of protein and demonstrated sequential improvement in the linearity of the signal at lower concentrations of protein (See Additional file 1: Figure S1). Thus we believe that the adjustment of protein quantity per capillary is an important factor to be considered when setting up these assays.

### Nano-immunoassay provides a reliable and sensitive measurement of 4EBP1 and Akt activation in primary patient samples

**Figure 6:**
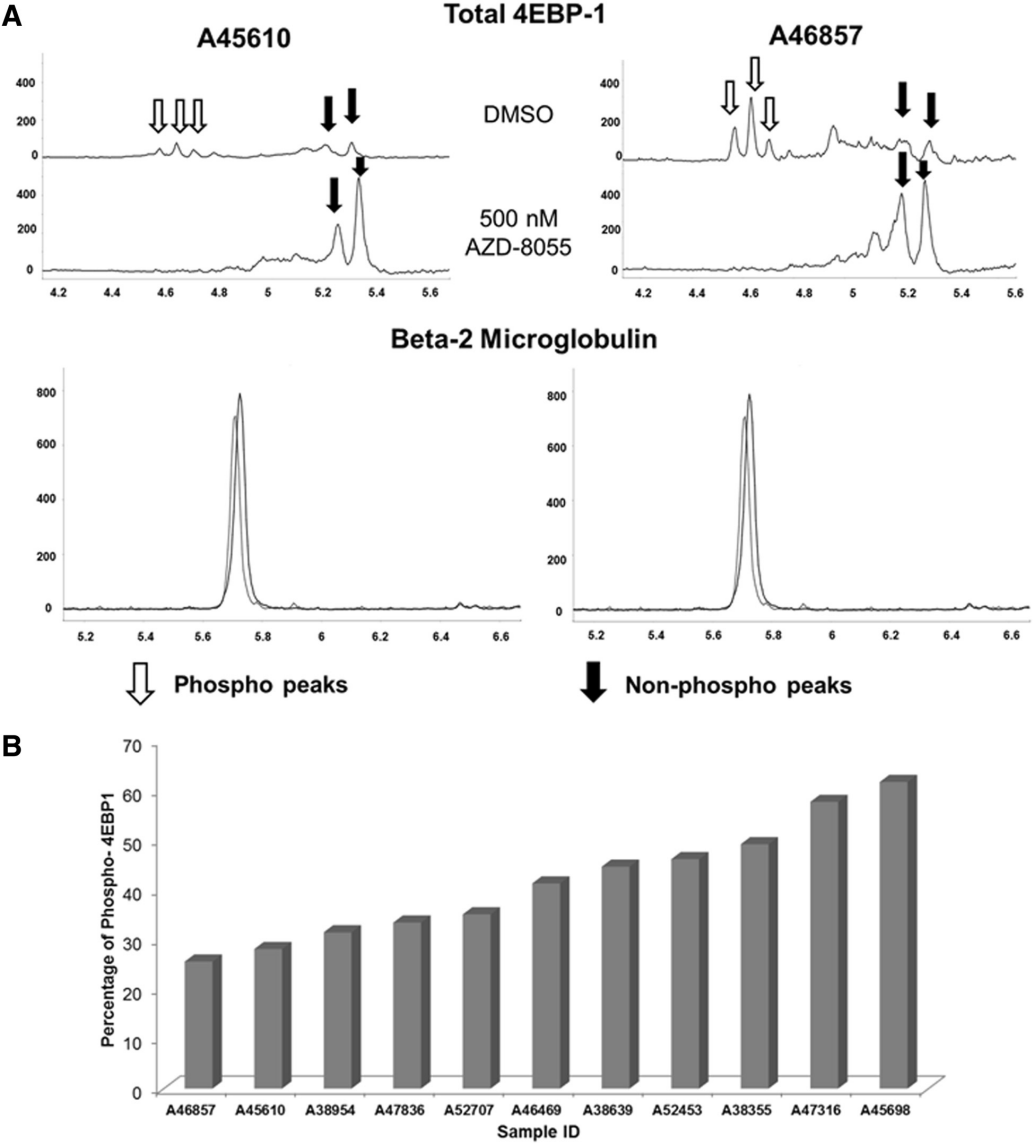
**Measurement of total and phosphorylated 4EBP1 in primary AML samples. A)** Samples (n = 8) were treated with 500 nM AZD-8055 and electropherogram demonstrated decrease in phosphorylated forms using total 4EBP1 antibody. Two representative samples are shown. β-2 Microglobulin was used as loading control. X-axis represents iso-electric pH and y-axis represents luminescence units. **B)** Primary AML patient samples were examined at baseline for total and phosphorylated forms of 4EBP1 and expressed as percentage phosphorylated against sample number (n = 11).

Primary pediatric bone marrow samples were obtained from patients enrolled on the Children’s Oncology Group trial AAML 0531. Samples were examined for baseline activation of 4EBP1 (Figure 6). A total of 1.6 × 10^6^ cells were lysed in 18 μl of lysis buffer and used for this baseline analysis which accounted for approximately 8888 cells per capillary. The signals obtained using the phosphorylated antibodies was extremely low so the signal from the Total 4EBP1 antibody was used for further analysis. The primary samples were treated with AZD-8055 (500 nM) for 24 hours and the changes in the peak profile were used to determine the phosphorylated peaks within samples (n = 8). Two representative samples are shown in Figure 6-A. Treatment with AZD-8055 resulted in a decrease in phosphorylated peaks and an increase in the un-phosphorylated more basic peaks. β-2 microglobulin was used as a loading control. A total of 11 samples were probed with the total 4EBP1 antibody and all antibody signals were amplified using streptavidin-biotin amplification reagent. We further used the AUC to calculate the percentage of phosphorylated 4EBP1 in each AML sample. The range of 4EBP1 phosphorylation varied widely from 25.5 – 61.5% (Figure 6-B). Since 4EBP1 is a ubiquitous and abundant protein, it is likely that phosphorylation is only transient within cells and might account for the wide range of activation seen in the primary samples.

**Figure 7:**
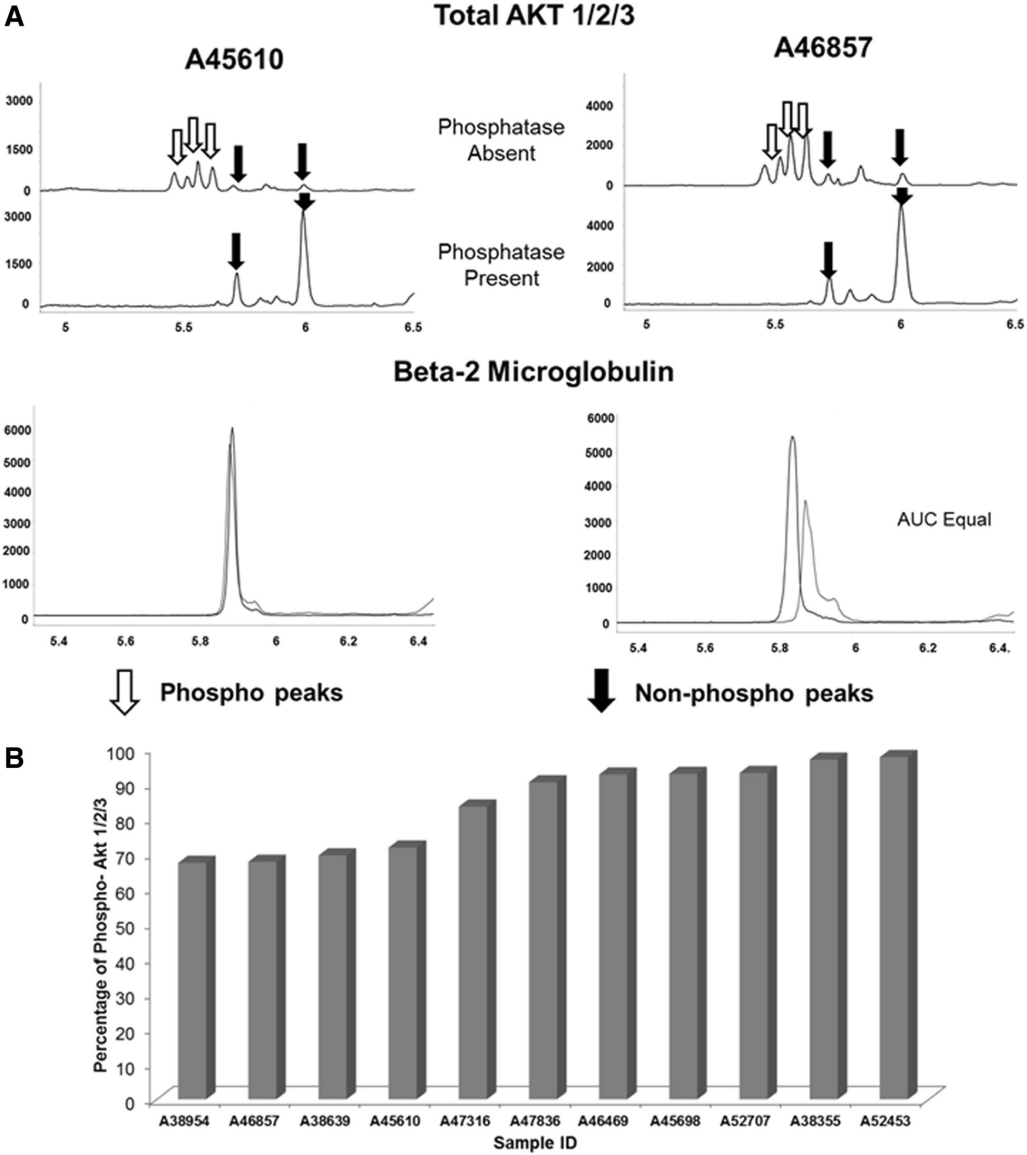
**Measurement of total and phosphorylated Akt 1/2/3 in primary AML samples. A)** Samples (n = 4) were treated with lambda phosphatase and electropherogram demonstrated decrease in phosphorylated forms using total Akt 1/2/3 antibody. Two representative samples are shown. β-2 Microglobulin was used as loading control. X-axis represents iso-electric pH and y-axis represents luminescence units. **B)** Primary AML patient samples were examined at baseline for total and phosphorylated forms of Akt 1/2/3 and expressed as percentage phosphorylated against sample number (n = 11).

Each sample was also analyzed for activation of Akt 1/2/3 using total Akt 1/2/3 antibody (Figure 7). In order to determine phosphorylated peaks, samples were treated with lambda phosphatase (n = 4) which showed a decrease in the acidic peaks with corresponding increase in the basic peaks as expected with inhibition of phosphorylation. Two representative samples are shown in Figure 7-A. β-2 microglobulin was used as a loading control. We used the AUC to further calculate the percentage of phosphorylated protein within each sample. As opposed to 4EBP1, Akt 1/2/3 showed higher degrees of phosphorylation ranging from 67.2 – 97.3% (n = 11). This may be likely due to the fact that 4EBP1 phosphorylation is transient whereas Akt is constitutively phosphorylated at certain sites [17].

**Figure 8:**
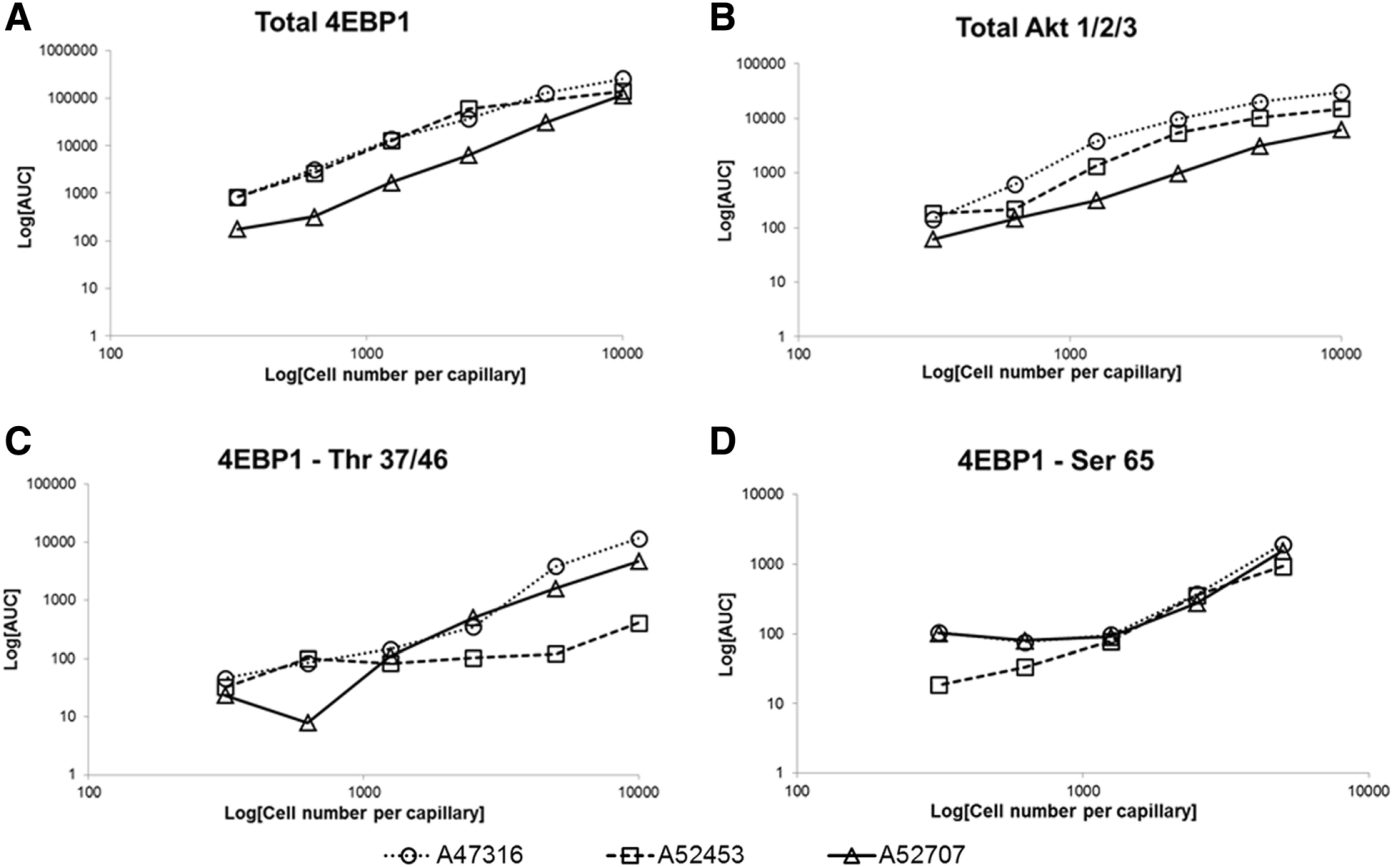
**The Nanopro 1000 is capable of detecting signal using low cell numbers in primary AML samples.** Three independent primary AML samples (A47316, A52453 and A52707) were used to test the capability of the assay to detect signal. Antibodies tested were Total 4EBP1 **(A)**, Total Akt 1/2/3 **(B)**, Phospho-4EBP1 Thr 37/46 **(C)** and Phospho-4EBP1 Ser 65 **(D)** Cell numbers ranged from 10,000-313 cells per capillary. Signal intensity (AUC, log-scale, y-axis) was plotted against cell numbers (log-scale, x-axis) and showed a linear increase with increasing cell numbers.

In order to determine the sensitivity of our assays in primary cells, we performed sequential dilution of the primary bone marrow samples (n = 3) and analyzed the signal with total and phosphorylated 4EBP1 antibodies and total Akt 1/2/3 antibody (Figure 8). A total of 16 × 10^6^ cells were lysed in 140 μl lysis buffer and protein concentrations were determined. Samples were run in duplicate on the assay plate at cell numbers ranging from 10,000 to 313 cells per capillary which corresponded to protein concentrations ranging from 370 ng to 5.9 ng of protein per capillary. It is important to note that protein concentrations can vary from sample to sample in spite of having similar cell numbers since it is dependent on factors such as percentage of blasts in bone marrow, size of blasts etc. The AUC for each antibody was plotted against cell numbers per capillary for each of the samples. A dose-dependent increase in signal was noted with increasing cell numbers (Figure 8A-D) with R-squared values >0.92 except for sample A52453 –Thr 37/46 4EBP1 with R-squared value of 0.88. Thus the assay was fairly robust over a wide range of protein concentrations (5.9 - 370 ng) and cell numbers (313–10,000) per capillary.

## Discussion

Acute myeloid leukemia continues to be a therapeutic challenge with low overall survival rates and high incidence of relapse. Development of improved biological correlates that define the disease may allow for risk stratification of patients that can be incorporated into current risk strata based on cytogenetic and molecular abnormalities. Defining the activation status of the 4EBP1 and Akt 1/2/3 proteins can serve as an important indicator of signal transduction in AML and can potentially provide information of prognostic significance. Using the nano-immunoassay platform we were able to standardize assays for both 4EBP1 and Akt 1/2/3 in AML cell lines.

Traditionally, Western blotting and intra-cellular flow cytometry have been used to study individual protein activation. However both of these techniques need a large number of cells, are unable to look at multiple sites in the same protein in the same assay, and in the case of flow cytometry require significant operator expertise. The NanoPro assays have the advantage of being automated and are amenable to clinical translation due to the rapid turn-around time and the routine methods for data acquisition. While not capable of providing the depth of information on single cells that can be achieved from flow cytometry analysis, the nano-immunoassay platform can be combined with flow cytometry sorting to characterize rare AML sub-populations. Enrichment of whole AML bone marrow or peripheral blood for important cell subsets such as CD34^+^CD38^-^ populations can be achieved by flow cytometry and subsequently these cells can be lysed and analyzed using the NanoPro platform. Multi-color flow cytometry confers the advantage of being able to study different phospho-proteins in gated populations but can also be limited by the number of phospho-proteins studied simultaneously (typically <12) and can be technically challenging. The NanoPro technology supplements flow cytometry with the ability to quantify phosphorylation patterns and examine other post-translational modifications such as acetylation and methylation. Since the separation of the protein is based on iso-electric pH, this platform can use total protein antibodies to determine multi-phosphorylation events as demonstrated here for 4EBP1 and Akt1/2/3 since heavily phosphorylated proteins tend to have lower iso-electric pH.

More recently, Reverse Phase Protein Array (RPPA) analysis has been used to study the effects of protein expression and modification in tumor samples [18]. RPPA is a high-throughput antibody based technique capable of screening a large number of antibodies in a single assay however our nano-immunoassay has certain advantages. Detection of multiple phosphorylation states of a protein using RPPA requires the use of multiple phospho-specific antibodies while in case of the nano-immunoassay, a single total antibody (eg. Akt 1/2/3) can provide accurate information about multiple phosphorylation sites. RPPA also has a much longer turn-around time of several weeks whereas the NanoPro can provide results within 24 hours making it a viable option for real-time analysis of patient samples.

Considering the above mentioned advantages of the NanoPro system, we standardized the assays for 4EBP1 and Akt 1/2/3. Depending on the antibody and the degree of expression, we were able to detect a signal for most antibodies with as low as 80 ng of protein per capillary and in certain cases (total 4EBP1) even as low as 20 ng per capillary. The assays were robust with a short turn-around time of 24 hours providing results that were quantitative and easy to interpret. We further tested these assays in primary bone marrow samples from pediatric AML patients and found the results to be consistent and reproducible. Primary cells are smaller than cell lines but we were able to reliably detect signal in as low as 40–96 ng of protein. Although the amount of protein needed for performing these assays per capillary is low, there are certain limiting factors that can affect the sample preparation that need consideration. Firstly, though cell counts for AML blasts might be high in samples, these cells are still fairly small in size as compared to AML cell lines. The recommended amount of protein per capillary is 80 ng which corresponds roughly to 2500 primary AML cells and ranged from 900–1400 cells from cultured AML cell lines. Secondly, in order to achieve these numbers, we had to lyse at least 1.6 million primary cells in lysis buffer to be able to obtain adequate signal for each antibody. Therefore, future studies that focus on leukemia stem cells or minimal residual disease would require incorporation of methods for concentrating protein as part of the sample preparation prior to running on the NanoPro.

Both 4EBP1 and Akt are proteins that are phosphorylated on multiple sites. Using the nano-immunoassay we were able to distinguish these multiple phosphorylated forms in cell lines and primary AML samples. We tested both AML cell lines and primary AML samples with AZD-8055 mTOR 1/2 inhibitor and found that the drug was effective at inhibiting 4EBP1 phosphorylation thus making this technology useful to determine specific target inhibition. We are also currently working on developing assays that cover the entire PI3K-Akt-mTOR pathway. Assays for PI3K activation or p70S6K activation have been problematic and require additional development. Therefore, it is possible that our assay could miss some p70S6K activation through the ERK signaling pathway. We could therefore combine the analysis of Akt 1/2/3 and 4EBP1 phosphorylation with analysis of pERK in future studies. The assay for pERK is well described by several groups in non-hematologic cancers [19] but thus far has not worked on AML samples in our laboratory.

Since this was a pilot study utilizing a small number of samples to highlight an emerging new technology, we were unable to make any relevant prognostic conclusions correlating signal strength to overall survival/relapse rates. Our future studies will involve studying larger numbers of patient samples with correlative outcome data as well as comparison of samples at diagnosis and relapse to determine changes in protein activation. The utility of our overall approach to study signal activation is broad and could apply not only to leukemia but also to other cancers where tumor samples might be limiting in number.

## Competing interests

The authors declare that they have no competing interests.

## Authors’ contributions

HSS and HLB performed the experiments. HSS, HLB, STB, TMC, and KDB analyzed the data. HSS and KDB drafted the manuscript. All authors read and approved the final manuscript.

## Supplementary Material

Additional file 1: Figure S1 **Protein amount is important for accurate measurement of signal.** The amount of protein per capillary was titrated (20–80 ng) in MV4-11, MOLM-14, OCI-AML3 and HL60 cell lines and increase in signal linearity was observed with improvement of R-squared values for total 4EBP1 antibody. Click here for file
