# Supplementary figures and images for: Capillary nano-immunoassay for Akt 1/2/3 and 4EBP1 phosphorylation in acute myeloid leukemia
Source: J Transl Med. 2014 Jun 12;12:166. doi: 10.1186/1479-5876-12-166 (PMC4080754; doi:10.1186/1479-5876-12-166)

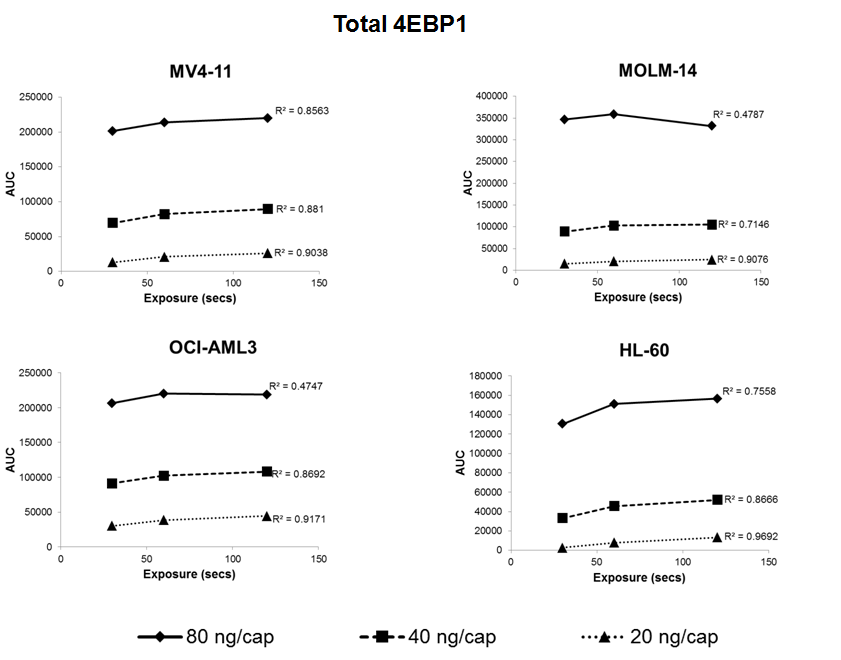

Supplement: Additional file 1: Figure S1 — Protein amount is important for accurate measurement of signal. The amount of protein per capillary was titrated (20–80 ng) in MV4-11, MOLM-14, OCI-AML3 and HL60 cell lines and increase in signal linearity was observed with improvement of R-squared values for total 4EBP1 antibody. [file 1479-5876-12-166-S1.tiff]
